# The Mind of a Bee

**DOI:** 10.1017/awf.2023.11

**Published:** 2023-03-01

**Authors:** Helen Gray

**Affiliations:** Newcastle University, UK

Content warnings: Mental health issues and suicide

So far in my relatively short career, I have researched the behaviour and welfare of rhesus macaques, pigs, and laying hens. However, my first taste of animal behaviour was as an undergraduate in a honeybee laboratory, training bees to respond to odours for sugar rewards. I was therefore delighted to be asked to review this book and to be given a chance to catch up on the latest in bee research.

The book starts with an overarching introduction in which the author gives a basic overview of bees, followed by a handy roadmap of the chapters and an explanation of the use of historical literature and biographical details of the scientists he cites (more on that later). Chittka ends by promising to take the reader on a “journey into the minds of bees.”

The following ten chapters (chapters 2–11) deal with bee sensory processing, instinct behaviours, communication, cognition, brain physiology, personality, and consciousness. Each chapter is further broken down into bitesize chunks of information which have been succinctly summarised and are easy to follow. A range of brilliant figures support each chapter and when dealing with the particulars of, for example, spatial cognition experiments and detailed brain anatomy, these are very welcome aides. Throughout, Chittka does an excellent job of combining historic and contemporary research to build an overall picture of the current state of knowledge for each theme, as well as identifying where the gaps remain. There is much to glean from this book on the basics of behaviour, the rules of learning, and comparative brain anatomy, to name but a few.

Complicated topics are dealt with in the book and, in my opinion, some clearer definitions of these would have been useful. The chapter covering personality for instance would have benefitted from a brief overview of what is considered as personality in different fields. Indeed, this would also have helped when issues such as culture, emotion and consciousness were discussed. Although the intricacies of these concepts are contentious and debated, more formal definitions in the text would have guided the reader to the specific meaning with which Chittka was aligning his views and evidence.

The final chapter (12), “What our knowledge of bees’ minds means for their conservation”, is written as an Afterword and is therefore very short. I was a little disappointed as this is the section which deals with the moral implications of our reliance on bees for agriculture, our duty to protect wild bees, and the ethical issues in using bees for research. As an animal welfare scientist, I was left wanting to know more about how the author sees the future of the more invasive laboratory studies. Maybe in the next book!

Aside from the breakdown of the science of bees, there are three things I appreciated in Chittka’s writing: 1) His acknowledgement of the ideas and contributions made by students and postdoctoral researchers; 2) providing historical context to the science, and 3) highlighting the personal struggles of the scientists as individuals. To expand on points two and three: Throughout the book, Chittka weaves in the history of the scholars whose science he discusses and the social context in which they performed their work. In doing so he highlights the crucial but much-forgotten experiments of African American ethologist Charles Turner and the challenging conditions under which Karl von Frisch conducted work as a scholar with Jewish heritage in Nazi Germany. Chittka also shines a light on the mental health issues faced by academics. He tells the stories of Frederick C Kenyon and Martin Hammer, decades apart, both of whom made huge contributions to their fields but struggled to find permanent employment, and who ultimately ended up confined to an asylum (Kenyon) and suspected to have taken their own life (Hammer). These stories, in my view, hold equal importance as the scientific contributions Kenyon and Hammer made. They are a reminder of the extreme pressure academics find themselves under and a prompt for us all to challenge the conditions which cause such losses.

Overall, the book is broad enough to be accessible to a lay audience whilst providing sufficient detail to be engaging for academics. Though I found myself wanting more in-depth explanations in certain parts (and had to remind myself it was not written to be a textbook), it still provides a springboard for anyone who wishes to further expand their knowledge. I would enthusiastically recommend this book as an entertaining and enlightening read.

